# Stomatis Materna

**Published:** 1856-06

**Authors:** 


### STOMATITIS MATERNA.

By reference to the proceedings of the American Medical Association, it will be perceived that a committee was appointed by that body to report upon this disease, and that one of the editors of this Journal was appointed chairman. As this disease, which is so rare in the East that many eminent medical men in large practice, in some of the Eastern Atlantic cities, have never seen a case of it, it is, however, of but too frequent occurrence in the West and therefore it becomes the duty of the profession here to institute enquiries into its pathology and the best remedial measures.

We would therefore, earnestly call upon our medical brethren to communicate to us all the information in their possession, touching its etilogy, pathology, symptoms, duration, the circumstances under which it has occurred, the treatment most successful, the termination, the temperament of constitution, the effects upon the offspring, with all other facts which may tend to throw light upon a subject about which so little comparatively has been written.

The "nursing sore mouth" has become so frequent in its occurrence, and the results so unfortunate in many instances, to both mother and child, that the enquiry is one of the greatest importance in medical science, and second to none in interest to the western practitioner. Every consideration of humanity demands that the profession should push forward its enquiries, until more is known of its pathology and the best remedies to combat it.

By reference to the pages of the 2d volume of this work, it will be seen that Dr. Knapp takes the position, that it is scorbutic in its character, with which opinion we are not disposed to coincide. We would be very glad if our medical brethren, in the progress of their investigations, would examine this question and report whether they have observed the similarity which he claims to exist between the diseases, or indeed, the identity which he urges obtains between scurvy and the *stomatitis materna.* We fear the Dr. is too much inclined to enrol more of the ills to which the human frame is incident under the head of "scurvy" than is justly entitled to such a classification.

